# The Cause

**Published:** 1896-01

**Authors:** Amelia A. Waterhouse

**Affiliations:** San Francisco, Cal.


### THE CAUSE.

Amelia A. Waterhouse, M. D., San Francisco, Cal.

What is the cause of paresis, rheumatism, constipation, and, I might add, innumerable diseases? Doctors say we cannot explain why there is so much paresis nowadays, why heart disease, why this and that; the cause is physic; let me explain. How is it if the blood is poor, and why is the blood poor? It is this: Nature knows how to make blood; we physic it; the good material is all gone, and what has Nature to do but succumb.

The blood is pumped from the heart into the arteries, then through the capillaries and glands; if the force is not enough, little particles of poor blood or sediment will be left in the glands; hence cancer, tumors, ovarian troubles, tonsillitis, and many more troubles from the glands; then the arteries do their work if Nature has a full supply of good blood; if not, the heart becomes weak, and all the internal organs have to suffer. There is no nourishment for the glands of the stomach, none for the thousand little glands of the bowels. Why dyspepsia? Why colic? Why hæmorrhoids? Physic is my answer. I go further: the blood has to nourish the nerves, and if the nerves are not nourished there is paralysis, paresis, nervous prostration. Let me picture a sample of many of my cases. A girl comes to me so tired, no life, everything she eats distresses her.

I ask her about her bowels, if she has constipation. Oh! yes, unless I take pills or something to relax my bowels. Well if you wish my medicine and advice I expect you to follow my instructions (sometimes I find they will do so, then I have good results. I give advice in a strong manner). Oh! but I get so bilious, she says. Are you any better for taking your life into your own hands? I ask. Are you not defrauding Nature, who knows how to give you good blood, and you physic it all away? Poor, puny girls are soon made strong and tell me they could never have believed it would make such a difference.

Then the nerves again. Boils, hives, the blood is not doing its work, the nerves are dying, and hence the troubles I speak of. I could write pages upon this subject, and yet, will this not suffice to set people to thinking?

